# A NECessary Workup: Exploring the Rare Histology of an Esophageal Tumor

**DOI:** 10.1002/ueg2.70056

**Published:** 2025-06-24

**Authors:** Ronald Koschny, Georg Martin Haag, Alexander Brobeil

**Affiliations:** ^1^ Department of Gastroenterology Interdisciplinary Endoscopy Center (IEZ) University Hospital Heidelberg Heidelberg Germany; ^2^ Department of Medical Oncology National Center for Tumor Diseases Heidelberg University Hospital Heidelberg Germany; ^3^ Institute of Pathology University Hospital Heidelberg Heidelberg Germany

**Keywords:** esophagus, malignancy, neuroendocrine carcinoma

A 64 years old patient with melena was transferred for re‐biopsy of a suspected adenocarcinoma of the esophagogastric junction (Siewert type II) within Barrett's esophagus (C0M2) with “indeterminate dysplasia” due to insufficient external tissue sampling. The patient's comorbidities (male sex, age, obesity, smoking) supported the suspicion of Barrett's carcinoma. Figure 1 shows the endoscopic appearance of a stenosing tumor stretching from 35 to 45 cm from the incisors (Z‐line at 43 cm). Computed tomography described a T3 tumor with an increased number of morphologically non‐suspicious locoregional lymph nodes.

**FIGURE 1:**
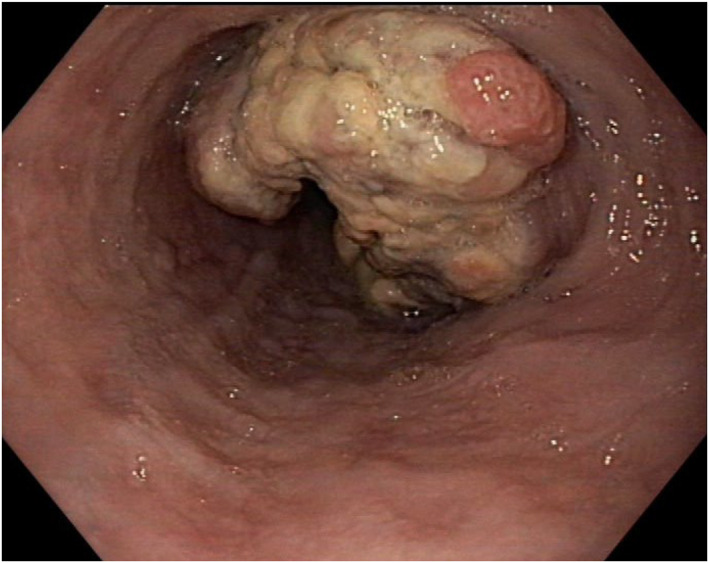
Endoscopic image of a stenosing tumor at the esophago‐gastric junction.

Histopathology of multiple re‐biopsies showed infiltrates of malignant cells positive for CD56 and synaptophysin confirming a neuroendocrine carcinoma (NEC) of the esophagus with a proliferation index (Ki67) of 90% (Figure 2). Expression of TTF1 and GATA3 raised the suspicion of a pulmonary primary. Serum chromogranin was not elevated.

**FIGURE 2:**
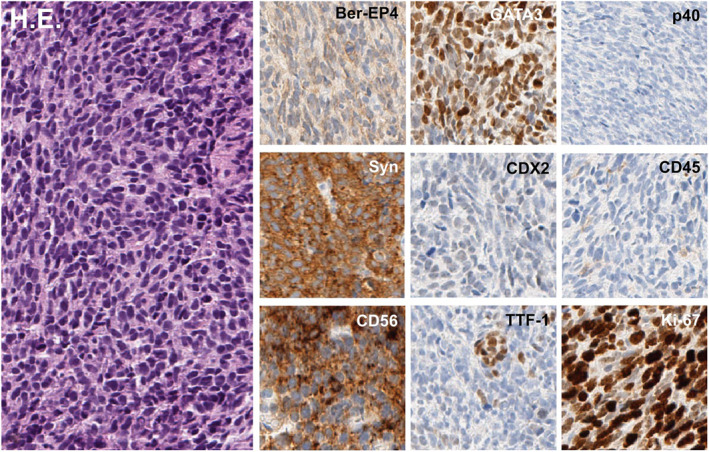
Hematoxylin‐eosin stain (left) and immunohistochemistry (right) of tumor samples.

After neoadjuvant treatment with two cycles of carboplatin/etoposide, the neuroendocrine carcinoma showed significant tumor shrinkage (Figure 3). The patient underwent abdomino‐thoracic esophagectomy (ypT1b ypN0 (0/11) L0 V0 G3 R0).

**FIGURE 3:**
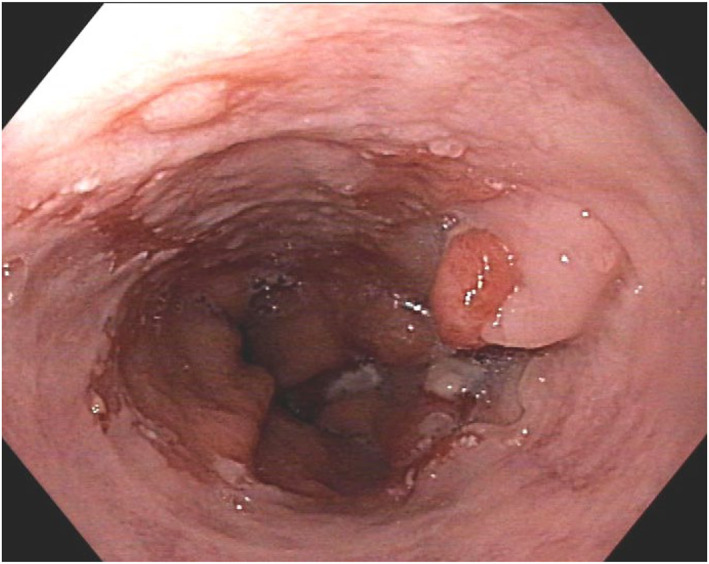
Endoscopic image after two cycles of carboplatin/etoposide.

Undergoing continuous monitoring by CT and endoscopy, no tumor relapse occurred during a 5‐year follow‐up. Initial CT scan and long‐term follow‐up excluded a pulmonary primary of the tumor.

Primary NEC of the esophagus accounts for only 3% of esophageal neoplasms [1]. Thus, NEC is a very rare differential diagnosis of tumors of the esophagogastric junction, especially when arising within Barrett's esophagus [2, 3]. Although their etiology is unknown, a common stem cell has been suggested due to the presence of endocrine cell hyperplasia in Barrett mucosa [4]. Alternatively, NEC might arise from hyperplasia of enterochromaffin‐like cells induced by long term proton pump inhibitor use in these patients [5].

Conclusive tumor histology cannot always be obtained from inflamed and necrotic tissue. However, even in putatively clinically conclusive situations, histological classification is an indispensable prerequisite for the correct tumor diagnosis and selection of the optimal neoadjuvant treatment. This case emphasizes the importance of repeated biopsies to avoid improper treatment.

## Conflicts of Interest

The authors declare no conflicts of interest.

## Data Availability

The data that support the findings of this study are available on request from the corresponding author. The data are not publicly available due to privacy or ethical restrictions.
